# Bis(2,6-dicarboxy­pyridinium) dichloride acetone monosolvate

**DOI:** 10.1107/S1600536809043220

**Published:** 2009-10-23

**Authors:** Cuong Quoc Ton, Michael Bolte

**Affiliations:** a Institut für Organische Chemie der Goethe-Universität Frankfurt, Max-von-Laue-Strasse 7, D-60438 Frankfurt am Main, Germany; b Institut für Anorganische Chemie der Goethe-Universität Frankfurt, Max-von-Laue-Strasse 7, D-60438 Frankfurt am Main, Germany

## Abstract

The title compound, 2C_7_H_6_NO_4_^+^·2Cl^−^·C_3_H_6_O, crystallizes with two 2,6-dicarboxy­pyridinium cations, two chloride anions and one acetone mol­ecule in the asymmetric unit. The crystal structure is characterized by alternating cations and by Cl^−^ anions, forming zigzag chains running along the *a* axis.

## Related literature

For co-crystallization experiments, see: Ton & Bolte (2005 ▶); Tutughamiarso *et al.* (2009 ▶).
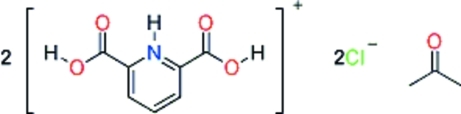


## Experimental

### 

#### Crystal data

2C_7_H_6_NO_4_^+^·2Cl^−^·C_3_H_6_O
*M* *_r_* = 465.23
Monoclinic, 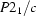

*a* = 21.108 (4) Å
*b* = 6.7877 (14) Å
*c* = 15.224 (3) Å
β = 110.28 (3)°
*V* = 2046.0 (7) Å^3^
*Z* = 4
Mo *K*α radiation
μ = 0.37 mm^−1^
*T* = 173 K
0.30 × 0.20 × 0.20 mm

#### Data collection

Stoe IPDSII two-circle diffractometer
Absorption correction: multi-scan (*MULABS*; Spek, 2003 ▶; Blessing, 1995 ▶) *T*_min_ = 0.897, *T*_max_ = 0.930
27731 measured reflections
3867 independent reflections
3412 reflections with *I* > 2σ(*I*)
*R*_int_ = 0.041

#### Refinement

*R*[*F* ^2^ > 2σ(*F* ^2^)] = 0.028
*wR*(*F* ^2^) = 0.082
*S* = 1.07
3867 reflections
277 parameters
2 restraints
H-atom parameters constrained
Δρ_max_ = 0.17 e Å^−3^
Δρ_min_ = −0.33 e Å^−3^

### 

Data collection: *X-AREA* (Stoe & Cie, 2001 ▶); cell refinement: *X-AREA*; data reduction: *X-AREA*; program(s) used to solve structure: *SHELXS97* (Sheldrick, 2008 ▶); program(s) used to refine structure: *SHELXL97* (Sheldrick, 2008 ▶); molecular graphics: *XP* in *SHELXTL* (Sheldrick, 2008 ▶); software used to prepare material for publication: *SHELXL97*.

## Supplementary Material

Crystal structure: contains datablocks I, global. DOI: 10.1107/S1600536809043220/ng2671sup1.cif

Structure factors: contains datablocks I. DOI: 10.1107/S1600536809043220/ng2671Isup2.hkl

Additional supplementary materials:  crystallographic information; 3D view; checkCIF report

## Figures and Tables

**Table 1:** Hydrogen-bond geometry (Å, °)

*D*—H⋯*A*	*D*—H	H⋯*A*	*D*⋯*A*	*D*—H⋯*A*
O2—H2⋯Cl2^i^	0.84	2.11	2.9469 (13)	171
O3—H3⋯Cl1^ii^	0.84	2.14	2.9727 (13)	172
O12—H12⋯Cl1	0.84	2.13	2.9696 (15)	179
O14—H14⋯Cl2	0.84	2.14	2.9775 (12)	177
N1—H1*N*⋯O30^iii^	0.88	2.42	3.277 (2)	166
N1—H1*N*⋯O2	0.88	2.34	2.6685 (16)	103
N1—H1*N*⋯O4	0.88	2.39	2.7195 (16)	103
N2—H2*N*⋯O11	0.88	2.25	2.6365 (17)	106
N2—H2*N*⋯O13	0.88	2.26	2.6392 (16)	106

Symmetry codes: (i) 
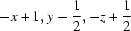
; (ii) 
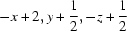
; (iii) 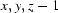.

**Fig. 1.**
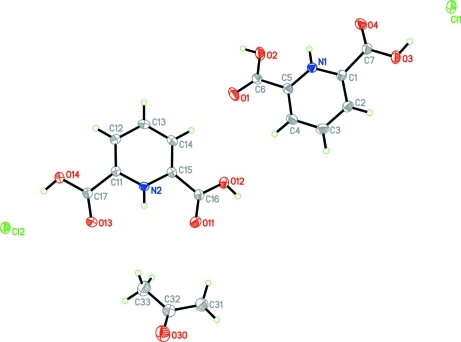
A view of the molecular structure of the title compound, with the atom-numbering scheme. Displacement ellipsoids are drawn at the 50% probability level and H atoms are shown as small spheres of arbitrary radii.

**Fig. 2.**
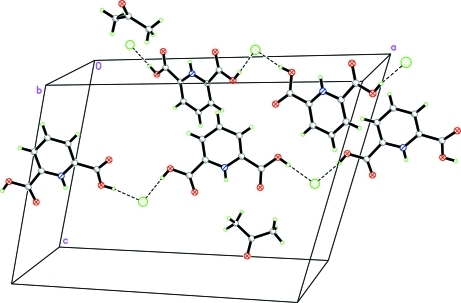
Part of the crystal packing of the title compound. Hydrogen bonds are shown as dashed lines.

## supplementary crystallographic information

### Comment

The aim of our research is the cocrystallization of two small organic compounds in order to examine the hydrogen bonds formed between hydrogen-bond acceptors and hydrogen-bond donors (Ton & Bolte, 2005; Tutughamiarso *et al.*, 2009). When pyridine-2,6-dicarbonyl dichlorid and resorcinol were mixed in order to obtain a hydrogen bonded supermolecular complex, it turned out that the pyridine-2,6-dicarbonyl dichlorid had been hydrolyzed to the dicarboxylic acid. The title compound crystallizes with two 2,6-dicarboxypyridinium cations, two chloride anions and one acetone molecule in the asymmetric unit. The crystal structure is characterized by alternating cations and by Cl^-^ anions forming zigzag chains running along the *a* axis. The amino H atoms do not form intermolecular hydrogen bonds, but show short distances to the O atoms of the adjacent carboxyl groups.

### Experimental

Pyridine-2,6-dicarbonyl dichlorid (20 mg) and resorcinol (20 mg) were dissolved in 2 ml absolute acetone. The mixture was sealed and set aside at room temperature. After two weeks small block-shaped crystals were obtained. It turned out that the pyridine-2,6-dicarbonyl dichloride had been hydrolyzed to the dicarboxylic acid.

### Refinement

Hydrogen atoms were located in a difference Fourier map but they were included in calculated positions [N—H = 0.88 Å, C—H = 0.93 - 0.99 Å] and refined as riding [*U*_iso_(H) = 1.2*U*_eq_(C) or *U*_iso_(H) = 1.5*U*_eq_(*O*,*C*_methyl_)].

### Crystal data

**Table:**

2C_7_H_6_NO_4_^+^·2Cl^−^·C_3_H_6_O	*F*(000) = 960
*M**_r_* = 465.23	*D*_x_ = 1.510 Mg m^−^^3^
Monoclinic, *P*2_1_/*c*	Mo *K*α radiation, λ = 0.71073 Å
Hall symbol: -P 2ybc	Cell parameters from 4736 reflections
*a* = 21.108 (4) Å	θ = 3.6–23.9°
*b* = 6.7877 (14) Å	µ = 0.37 mm^−^^1^
*c* = 15.224 (3) Å	*T* = 173 K
β = 110.28 (3)°	Block, colourless
*V* = 2046.0 (7) Å^3^	0.30 × 0.20 × 0.20 mm
*Z* = 4	

### Data collection

**Table:**

Stoe IPDSII two-circle diffractometer	3867 independent reflections
Radiation source: fine-focus sealed tube	3412 reflections with *I* > 2σ(*I*)
graphite	*R*_int_ = 0.041
ω scans	θ_max_ = 25.7°, θ_min_ = 2.7°
Absorption correction: multi-scan (*MULABS*; Spek, 2003; Blessing, 1995)	*h* = −25→25
*T*_min_ = 0.897, *T*_max_ = 0.930	*k* = −8→8
27731 measured reflections	*l* = −18→18

### Refinement

**Table:**

Refinement on *F*^2^	Primary atom site location: structure-invariant direct methods
Least-squares matrix: full	Secondary atom site location: difference Fourier map
*R*[*F*^2^ > 2σ(*F*^2^)] = 0.028	Hydrogen site location: inferred from neighbouring sites
*wR*(*F*^2^) = 0.082	H-atom parameters constrained
*S* = 1.07	*w* = 1/[σ^2^(*F*_o_^2^) + (0.0611*P*)^2^] where *P* = (*F*_o_^2^ + 2*F*_c_^2^)/3
3867 reflections	(Δ/σ)_max_ = 0.001
277 parameters	Δρ_max_ = 0.17 e Å^−^^3^
2 restraints	Δρ_min_ = −0.33 e Å^−^^3^

### Special details

**Table:**

Geometry. All e.s.d.'s (except the e.s.d. in the dihedral angle between two l.s. planes) are estimated using the full covariance matrix. The cell e.s.d.'s are taken into account individually in the estimation of e.s.d.'s in distances, angles and torsion angles; correlations between e.s.d.'s in cell parameters are only used when they are defined by crystal symmetry. An approximate (isotropic) treatment of cell e.s.d.'s is used for estimating e.s.d.'s involving l.s. planes.
Refinement. Refinement of *F*^2^ against ALL reflections. The weighted *R*-factor *wR* and goodness of fit *S* are based on *F*^2^, conventional *R*-factors *R* are based on *F*, with *F* set to zero for negative *F*^2^. The threshold expression of *F*^2^ > σ(*F*^2^) is used only for calculating *R*-factors(gt) *etc*. and is not relevant to the choice of reflections for refinement. *R*-factors based on *F*^2^ are statistically about twice as large as those based on *F*, and *R*- factors based on ALL data will be even larger.

### Fractional atomic coordinates and isotropic or equivalent isotropic displacement parameters (Å^2^)

**Table:**

	*x*	*y*	*z*	*U*_iso_*/*U*_eq_	
Cl2	0.400074 (15)	0.86812 (5)	0.57272 (2)	0.02093 (10)	
N1	0.86727 (6)	0.62429 (16)	0.08751 (8)	0.0176 (2)	
H1N	0.8461	0.6094	0.0269	0.021*	
O1	0.72464 (5)	0.5375 (2)	0.15456 (8)	0.0373 (3)	
O2	0.74328 (5)	0.47101 (18)	0.02079 (7)	0.0314 (3)	
H2	0.7020	0.4421	0.0003	0.047*	
O3	1.02593 (5)	0.77466 (16)	0.07980 (7)	0.0269 (2)	
H3	1.0420	0.7892	0.0369	0.040*	
O4	0.93315 (5)	0.66689 (17)	−0.03641 (7)	0.0312 (3)	
C1	0.93284 (6)	0.67693 (19)	0.11849 (9)	0.0185 (3)	
C2	0.96772 (7)	0.6960 (2)	0.21350 (10)	0.0225 (3)	
H2A	1.0144	0.7289	0.2358	0.027*	
C3	0.93390 (8)	0.6665 (2)	0.27580 (10)	0.0259 (3)	
H3A	0.9572	0.6793	0.3413	0.031*	
C4	0.86538 (7)	0.6179 (2)	0.24171 (10)	0.0234 (3)	
H4	0.8412	0.6016	0.2835	0.028*	
C5	0.83310 (7)	0.59365 (19)	0.14648 (9)	0.0194 (3)	
C6	0.76021 (7)	0.5313 (2)	0.10756 (10)	0.0224 (3)	
C7	0.96393 (7)	0.7065 (2)	0.04423 (9)	0.0210 (3)	
C11	0.55437 (6)	0.66485 (18)	0.39734 (9)	0.0170 (3)	
C12	0.52686 (7)	0.65541 (19)	0.30102 (9)	0.0194 (3)	
H12A	0.4804	0.6830	0.2695	0.023*	
C13	0.56847 (7)	0.6047 (2)	0.25084 (9)	0.0220 (3)	
H13	0.5505	0.6010	0.1844	0.026*	
C14	0.63629 (7)	0.5591 (2)	0.29749 (9)	0.0202 (3)	
H14A	0.6647	0.5235	0.2635	0.024*	
C15	0.66136 (6)	0.56671 (19)	0.39429 (9)	0.0173 (3)	
C16	0.73154 (6)	0.51806 (19)	0.45933 (9)	0.0201 (3)	
C17	0.51949 (6)	0.71815 (19)	0.46516 (9)	0.0189 (3)	
N2	0.62003 (6)	0.62153 (15)	0.43989 (7)	0.0162 (2)	
H2N	0.6370	0.6296	0.5014	0.019*	
O11	0.74741 (5)	0.55571 (16)	0.54219 (7)	0.0281 (2)	
O12	0.76850 (5)	0.43135 (16)	0.41724 (7)	0.0260 (2)	
H12	0.8064	0.4033	0.4568	0.039*	
O13	0.54906 (5)	0.69891 (16)	0.54842 (7)	0.0269 (2)	
O14	0.45750 (5)	0.78176 (16)	0.42371 (7)	0.0244 (2)	
H14	0.4400	0.8075	0.4643	0.037*	
O30	0.77087 (6)	0.63026 (17)	0.86620 (9)	0.0391 (3)	
C31	0.81624 (8)	0.4065 (2)	0.78336 (11)	0.0325 (3)	
H31A	0.8598	0.4523	0.8271	0.049*	
H31B	0.8139	0.4325	0.7190	0.049*	
H31C	0.8118	0.2646	0.7917	0.049*	
C32	0.76014 (7)	0.5135 (2)	0.80203 (11)	0.0271 (3)	
C33	0.68937 (8)	0.4701 (3)	0.73731 (13)	0.0398 (4)	
H33A	0.6597	0.4538	0.7741	0.060*	
H33B	0.6893	0.3488	0.7024	0.060*	
H33C	0.6730	0.5797	0.6933	0.060*	
Cl1	0.903216 (16)	0.33503 (5)	0.55619 (2)	0.02480 (11)	

### Atomic displacement parameters (Å^2^)

**Table:**

	*U*^11^	*U*^22^	*U*^33^	*U*^12^	*U*^13^	*U*^23^
Cl2	0.01679 (17)	0.02369 (18)	0.02316 (18)	0.00153 (11)	0.00802 (13)	0.00127 (12)
N1	0.0179 (5)	0.0192 (5)	0.0153 (5)	0.0008 (4)	0.0052 (4)	−0.0010 (4)
O1	0.0271 (6)	0.0621 (8)	0.0297 (6)	−0.0071 (5)	0.0188 (5)	−0.0038 (5)
O2	0.0185 (5)	0.0508 (7)	0.0265 (6)	−0.0076 (5)	0.0098 (4)	−0.0110 (5)
O3	0.0157 (5)	0.0409 (6)	0.0248 (5)	−0.0049 (4)	0.0080 (4)	−0.0054 (4)
O4	0.0253 (5)	0.0496 (7)	0.0185 (5)	−0.0122 (5)	0.0074 (4)	−0.0023 (4)
C1	0.0176 (6)	0.0170 (6)	0.0206 (7)	0.0017 (5)	0.0064 (5)	−0.0001 (5)
C2	0.0196 (6)	0.0250 (7)	0.0207 (7)	0.0006 (5)	0.0042 (5)	−0.0020 (5)
C3	0.0285 (7)	0.0303 (7)	0.0164 (7)	0.0018 (6)	0.0047 (6)	−0.0015 (5)
C4	0.0275 (7)	0.0262 (7)	0.0191 (7)	0.0017 (5)	0.0115 (6)	0.0005 (5)
C5	0.0215 (7)	0.0174 (6)	0.0214 (7)	0.0026 (5)	0.0100 (5)	0.0012 (5)
C6	0.0222 (7)	0.0246 (7)	0.0222 (7)	0.0008 (5)	0.0101 (5)	0.0018 (5)
C7	0.0179 (6)	0.0233 (7)	0.0215 (7)	−0.0013 (5)	0.0065 (5)	−0.0008 (5)
C11	0.0175 (6)	0.0152 (6)	0.0188 (6)	−0.0004 (5)	0.0071 (5)	−0.0003 (5)
C12	0.0192 (6)	0.0187 (6)	0.0184 (6)	−0.0003 (5)	0.0039 (5)	0.0011 (5)
C13	0.0278 (7)	0.0220 (7)	0.0156 (6)	−0.0015 (5)	0.0070 (5)	0.0001 (5)
C14	0.0242 (7)	0.0201 (6)	0.0194 (6)	−0.0006 (5)	0.0117 (5)	−0.0003 (5)
C15	0.0186 (6)	0.0146 (6)	0.0202 (6)	−0.0011 (5)	0.0087 (5)	−0.0005 (5)
C16	0.0196 (6)	0.0196 (6)	0.0219 (7)	0.0005 (5)	0.0082 (5)	−0.0003 (5)
C17	0.0178 (6)	0.0201 (7)	0.0193 (6)	0.0007 (5)	0.0071 (5)	−0.0005 (5)
N2	0.0176 (5)	0.0174 (5)	0.0132 (5)	0.0004 (4)	0.0047 (4)	−0.0013 (4)
O11	0.0214 (5)	0.0392 (6)	0.0211 (5)	0.0048 (4)	0.0038 (4)	−0.0057 (4)
O12	0.0197 (5)	0.0340 (6)	0.0248 (5)	0.0084 (4)	0.0083 (4)	0.0002 (4)
O13	0.0241 (5)	0.0399 (6)	0.0174 (5)	0.0069 (4)	0.0081 (4)	−0.0004 (4)
O14	0.0188 (5)	0.0342 (6)	0.0217 (5)	0.0062 (4)	0.0091 (4)	0.0019 (4)
O30	0.0382 (6)	0.0362 (6)	0.0452 (7)	−0.0022 (5)	0.0175 (5)	−0.0108 (5)
C31	0.0323 (8)	0.0324 (8)	0.0326 (8)	0.0014 (6)	0.0111 (7)	−0.0022 (7)
C32	0.0305 (7)	0.0229 (7)	0.0288 (8)	−0.0012 (6)	0.0115 (6)	0.0049 (6)
C33	0.0292 (8)	0.0443 (10)	0.0429 (10)	0.0012 (7)	0.0087 (7)	−0.0006 (8)
Cl1	0.01629 (17)	0.02919 (19)	0.0289 (2)	0.00156 (12)	0.00780 (14)	−0.00016 (13)

### Geometric parameters (Å, °)

**Table:**

N1—C1	1.3467 (17)	C13—C14	1.394 (2)
N1—C5	1.3484 (18)	C13—H13	0.9500
N1—H1N	0.8800	C14—C15	1.3834 (19)
O1—C6	1.2040 (18)	C14—H14A	0.9500
O2—C6	1.3087 (18)	C15—N2	1.3421 (17)
O2—H2	0.8400	C15—C16	1.5057 (18)
O3—C7	1.3147 (17)	C16—O11	1.2149 (17)
O3—H3	0.8400	C16—O12	1.3085 (17)
O4—C7	1.2036 (17)	C17—O13	1.2103 (17)
C1—C2	1.3829 (19)	C17—O14	1.3132 (16)
C1—C7	1.5045 (19)	N2—H2N	0.8800
C2—C3	1.385 (2)	O12—H12	0.8400
C2—H2A	0.9500	O14—H14	0.8400
C3—C4	1.396 (2)	O30—C32	1.2167 (19)
C3—H3A	0.9500	C31—C32	1.498 (2)
C4—C5	1.381 (2)	C31—H31A	0.9800
C4—H4	0.9500	C31—H31B	0.9800
C5—C6	1.5052 (19)	C31—H31C	0.9800
C11—N2	1.3433 (17)	C32—C33	1.506 (2)
C11—C12	1.3790 (19)	C33—H33A	0.9800
C11—C17	1.5052 (18)	C33—H33B	0.9800
C12—C13	1.392 (2)	C33—H33C	0.9800
C12—H12A	0.9500		
			
C1—N1—C5	121.98 (11)	C14—C13—H13	119.8
C1—N1—H1N	119.0	C15—C14—C13	118.71 (12)
C5—N1—H1N	119.0	C15—C14—H14A	120.6
C6—O2—H2	109.5	C13—C14—H14A	120.6
C7—O3—H3	109.5	N2—C15—C14	118.96 (12)
N1—C1—C2	120.07 (13)	N2—C15—C16	112.85 (11)
N1—C1—C7	115.80 (12)	C14—C15—C16	128.19 (12)
C2—C1—C7	124.11 (12)	O11—C16—O12	127.34 (12)
C1—C2—C3	119.26 (13)	O11—C16—C15	119.41 (12)
C1—C2—H2A	120.4	O12—C16—C15	113.22 (12)
C3—C2—H2A	120.4	O13—C17—O14	127.23 (12)
C2—C3—C4	119.53 (13)	O13—C17—C11	119.68 (12)
C2—C3—H3A	120.2	O14—C17—C11	113.10 (11)
C4—C3—H3A	120.2	C15—N2—C11	123.93 (11)
C5—C4—C3	119.25 (13)	C15—N2—H2N	118.0
C5—C4—H4	120.4	C11—N2—H2N	118.0
C3—C4—H4	120.4	C16—O12—H12	109.5
N1—C5—C4	119.83 (12)	C17—O14—H14	109.5
N1—C5—C6	119.40 (12)	C32—C31—H31A	109.5
C4—C5—C6	120.77 (13)	C32—C31—H31B	109.5
O1—C6—O2	127.04 (13)	H31A—C31—H31B	109.5
O1—C6—C5	121.27 (13)	C32—C31—H31C	109.5
O2—C6—C5	111.69 (12)	H31A—C31—H31C	109.5
O4—C7—O3	127.38 (13)	H31B—C31—H31C	109.5
O4—C7—C1	120.99 (12)	O30—C32—C31	121.94 (14)
O3—C7—C1	111.63 (12)	O30—C32—C33	121.25 (15)
N2—C11—C12	119.10 (12)	C31—C32—C33	116.81 (14)
N2—C11—C17	112.96 (11)	C32—C33—H33A	109.5
C12—C11—C17	127.94 (12)	C32—C33—H33B	109.5
C11—C12—C13	118.80 (12)	H33A—C33—H33B	109.5
C11—C12—H12A	120.6	C32—C33—H33C	109.5
C13—C12—H12A	120.6	H33A—C33—H33C	109.5
C12—C13—C14	120.46 (12)	H33B—C33—H33C	109.5
C12—C13—H13	119.8		
			
C5—N1—C1—C2	1.76 (19)	N2—C11—C12—C13	−1.17 (19)
C5—N1—C1—C7	−179.77 (12)	C17—C11—C12—C13	179.30 (12)
N1—C1—C2—C3	−2.1 (2)	C11—C12—C13—C14	1.71 (19)
C7—C1—C2—C3	179.60 (13)	C12—C13—C14—C15	−0.4 (2)
C1—C2—C3—C4	0.1 (2)	C13—C14—C15—N2	−1.37 (19)
C2—C3—C4—C5	2.1 (2)	C13—C14—C15—C16	177.90 (12)
C1—N1—C5—C4	0.54 (19)	N2—C15—C16—O11	−9.64 (18)
C1—N1—C5—C6	−178.85 (11)	C14—C15—C16—O11	171.05 (13)
C3—C4—C5—N1	−2.5 (2)	N2—C15—C16—O12	168.43 (11)
C3—C4—C5—C6	176.92 (12)	C14—C15—C16—O12	−10.9 (2)
N1—C5—C6—O1	−166.16 (14)	N2—C11—C17—O13	−7.56 (18)
C4—C5—C6—O1	14.5 (2)	C12—C11—C17—O13	172.00 (13)
N1—C5—C6—O2	14.67 (18)	N2—C11—C17—O14	172.82 (11)
C4—C5—C6—O2	−164.72 (13)	C12—C11—C17—O14	−7.62 (19)
N1—C1—C7—O4	−6.70 (19)	C14—C15—N2—C11	2.01 (19)
C2—C1—C7—O4	171.71 (14)	C16—C15—N2—C11	−177.37 (11)
N1—C1—C7—O3	174.26 (11)	C12—C11—N2—C15	−0.71 (19)
C2—C1—C7—O3	−7.33 (19)	C17—C11—N2—C15	178.89 (11)

### Hydrogen-bond geometry (Å, °)

**Table:**

*D*—H···*A*	*D*—H	H···*A*	*D*···*A*	*D*—H···*A*
O2—H2···Cl2^i^	0.84	2.11	2.9469 (13)	171
O3—H3···Cl1^ii^	0.84	2.14	2.9727 (13)	172
O12—H12···Cl1	0.84	2.13	2.9696 (15)	179
O14—H14···Cl2	0.84	2.14	2.9775 (12)	177
N1—H1N···O30^iii^	0.88	2.42	3.277 (2)	166
N1—H1N···O2	0.88	2.34	2.6685 (16)	103
N1—H1N···O4	0.88	2.39	2.7195 (16)	103
N2—H2N···O11	0.88	2.25	2.6365 (17)	106
N2—H2N···O13	0.88	2.26	2.6392 (16)	106

Symmetry codes: (i) −*x*+1, *y*−1/2, −*z*+1/2; (ii) −*x*+2, *y*+1/2, −*z*+1/2; (iii) *x*, *y*, *z*−1.
